# *BARD1* Pathogenic Variants Are Associated with Triple-Negative Breast Cancer in a Spanish Hereditary Breast and Ovarian Cancer Cohort

**DOI:** 10.3390/genes12020150

**Published:** 2021-01-23

**Authors:** Paula Rofes, Jesús Del Valle, Sara Torres-Esquius, Lídia Feliubadaló, Agostina Stradella, José Marcos Moreno-Cabrera, Adriana López-Doriga, Elisabet Munté, Rafael De Cid, Olga Campos, Raquel Cuesta, Álex Teulé, Èlia Grau, Judit Sanz, Gabriel Capellá, Orland Díez, Joan Brunet, Judith Balmaña, Conxi Lázaro

**Affiliations:** 1 Hereditary Cancer Program, Catalan Institute of Oncology, IDIBELL, 08908 L’Hospitalet de Llobregat, Spain; profes@iconcologia.net (P.R.); jdelvalle@iconcologia.net (J.D.V.); lfeliubadalo@iconcologia.net (L.F.); astradella@iconcologia.net (A.S.); jmoreno@igtp.cat (J.M.M.-C.); emunte@iconcologia.net (E.M.); ocampos@iconcologia.net (O.C.); rcuesta@iconcologia.net (R.C.); ateule@iconcologia.net (Á.T.); gcapella@iconcologia.net (G.C.); 2 Program in Molecular Mechanisms and Experimental Therapy in Oncology (Oncobell), IDIBELL, 08908 L’Hospitalet de Llobregat, Spain; eggarces@iconcologia.net; 3 Centro de Investigación Biomédica en Red de Cáncer (CIBERONC), 28929 Madrid, Spain; 4 Hereditary Cancer Genetics Group, Vall d’Hebron Institute of Oncology (VHIO), Medical Oncology Department, University Hospital Vall d’Hebron, Universitat Autònoma de Barcelona, 08035 Barcelona, Spain; storres@vhio.net (S.T.-E.); jbalmana@vhio.net (J.B.); 5 Medical Oncology Department, Catalan Institute of Oncology, IDIBELL, 08908 L’Hospitalet de Llobregat, Spain; jbrunet@iconcologia.net; 6 Oncology Data Analytics Program (ODAP), Catalan Institute of Oncology, 08908 L’Hospitalet de Llobregat, Spain; alguerra@iconcologia.net; 7 Consortium for Biomedical Research in Epidemiology and Public Health (CIBERESP), 28029 Madrid, Spain; 8 Genomes for Life-GCAT Lab Group, IGTP, Institut Germans Trias i Pujol (IGTP), 08916 Badalona, Spain; rdecid@igtp.cat; 9 Hereditary Cancer Program, Catalan Institute of Oncology, IGTP, 08916 Badalona, Spain; 10 Genetic Counselling Unit, Medical Oncology Department, Althaia Xarxa Assistencial Universitària de Manresa, 08243 Manresa, Spain; jsanz@althaia.cat; 11 Catalan Health Institute, Vall d’Hebron Hospital Universitari, 08035 Barcelona, Spain; odiez@vhio.net; 12 Hereditary Cancer Genetics Group, Vall d’Hebron Institute of Oncology (VHIO), 08035 Barcelona, Spain; 13 Hereditary Cancer Program, Catalan Institute of Oncology, IDIBGI, 17007 Girona, Spain; 14 Medical Sciences Department, School of Medicine, University of Girona, 17007 Girona, Spain

**Keywords:** *BARD1*, breast cancer, triple-negative breast cancer, ovarian cancer, hereditary breast and ovarian cancer, moderate cancer risk

## Abstract

Only a small fraction of hereditary breast and/or ovarian cancer (HBOC) cases are caused by germline variants in the high-penetrance breast cancer 1 and 2 genes (*BRCA1* and *BRCA2)*. BRCA1-associated ring domain 1 (*BARD1*), nuclear partner of *BRCA1*, has been suggested as a potential HBOC risk gene, although its prevalence and penetrance are variable according to populations and type of tumor. We aimed to investigate the prevalence of *BARD1* truncating variants in a cohort of patients with clinical suspicion of HBOC. A comprehensive *BARD1* screening by multigene panel analysis was performed in 4015 unrelated patients according to our regional guidelines for genetic testing in hereditary cancer. In addition, 51,202 Genome Aggregation Database (gnomAD) non-Finnish, non-cancer European individuals were used as a control population. In our patient cohort, we identified 19 patients with heterozygous *BARD1* truncating variants (0.47%), whereas the frequency observed in the gnomAD controls was 0.12%. We found a statistically significant association of truncating *BARD1* variants with overall risk (odds ratio (OR) = 3.78; CI = 2.10–6.48; *p* = 1.16 × 10^−5^). This association remained significant in the hereditary breast cancer (HBC) group (OR = 4.18; CI = 2.10–7.70; *p* = 5.45 × 10^−5^). Furthermore, deleterious *BARD1* variants were enriched among triple-negative BC patients (OR = 5.40; CI = 1.77–18.15; *p* = 0.001) compared to other BC subtypes. Our results support the role of *BARD1* as a moderate penetrance BC predisposing gene and highlight a stronger association with triple-negative tumors.

## 1. Introduction

Hereditary breast and ovarian cancer (HBOC) risk has been traditionally linked to germline pathogenic variants (PVs) in breast cancer 1 and 2 genes (*BRCA1* and *BRCA2*). However, only 20–30% of high-risk families carry PVs in these genes [1]. Gradually, PVs in various other genes with different degrees of penetrance have also been associated with breast cancer (BC) and/or ovarian cancer (OC) risk [2]. Several genes that are either interacting with *BRCA1/2* or involved in DNA damage response pathways have also emerged as potential candidates that may account for some of the missing heritability of these so-called BRCAX families, although their associated risks have not been fully established [2].

BRCA1-associated ring domain 1 *(BARD1*) was first discovered in 1996 as the nuclear partner of BRCA1 and became one of the earliest candidates investigated [3]. It is localized on chromosome 2 at position 2q35 and encodes a protein of 777 amino acids that contains one N-terminal Really Interesting New Gene (RING)-finger domain, four Ankyrin (Ank) repeats and two C-terminal tandem BRCA1 C Terminus (BRCT) domains [4,5]. BARD1 shows structural homology with BRCA1 and they directly interact through their RING domains. The BARD1-BRCA1 obligate heterodimer functions as both an E3 ubiquitin ligase and as a direct mediator of homologous recombination for the recruitment of RAD51 to the sites of DNA double-strand break (DSB) [3,6,7]. Furthermore, BARD1 is also involved in other BRCA1-independent functions, including p53-mediated apoptosis [8]. 

To date, the role of *BARD1* in cancer predisposition remains inconclusive. Several case-control studies have reported a higher prevalence of deleterious *BARD1* variants among BC patients, supporting its role as a moderate risk predisposing gene [9,10,11]. An enrichment of *BARD1* PVs among triple-negative breast cancer (TNBC) cases has also been evidenced [12,13,14]. Contrarily, some studies have been unable to detect a significant association of *BARD1* with breast cancer risk [15,16]. Likewise, the association between *BARD1* and overall OC risk has shown controversial results [17,18,19]. Taken together, there is still insufficient evidence to elucidate the role of *BARD1* in breast and/or ovarian cancer predisposition. In the present study, we have investigated the prevalence of deleterious germline *BARD1* variants in a cohort of 4015 patients with clinical suspicion of hereditary breast and/or ovarian cancer, with the aim of elucidating the role of *BARD1* in cancer predisposition in the Spanish population.

## 2. Materials and Methods

### 2.1. Patients and Controls

A total of 4015 index patients with a personal or family history suggestive of hereditary BC and/or OC referring at genetic counseling units of the Catalan Institute of Oncology (ICO) and Vall d’Hebron (HVH) hospitals were included in the present study. Clinical characteristics for all enrolled patients were the following: patients with BC before 40 years; patients with TNBC before 60 years; male BC patients; patients with non-mucinous OC; patients with a family history of two cases of BC before age 50; patients with three or more cases of first-degree BC; patients with a case of bilateral BC associated with another case of BC in the family. Informed written consent for both diagnostic and research purposes was obtained from all patients, and the study protocol was approved by the ethics committee of Bellvitge Biomedical Research Institute (IDIBELL; PR278/19) and Vall d’Hebron Hospital (PRAG102-2016). A set of 194 Spanish cancer-free individuals from the Genomes For Life—Cohort Study of the Genomes of Catalonia (GCAT) cohort [20] were screened with the same cancer panel as ICO patients.

### 2.2. NGS Panel Testing

In the ICO cohort, genetic testing was performed on genomic DNA using the next-generation sequencing (NGS) custom panel I2HCP, which comprises 122–135 hereditary cancer (HC)-associated genes, depending on the version used [21]. Copy number analysis was performed from NGS data using DECoN [22] with parameter optimization [23]. Copy number variants (CNVs) in *BARD1* were validated using custom multiplex ligation-dependent probe amplification (MLPA) probes designed according to the instructions provided by MRC-Holland. Likewise, 26 HC-associated genes were included in the HVH NGS panel (BRCA Hereditary Cancer MASTR Plus kit, Agilent Technologies, Santa Clara, CA, USA). Copy number analysis was performed from NGS data using MASTR Reporter (Agilent Technologies, Santa Clara, CA, USA) and putative CNVs were validated by RT-PCR analysis [24]. For this study, we considered any variant that originates a premature stop codon or affects canonical splice site positions (+1,+2,−1,−2) as a pathogenic or likely pathogenic variant (pathogenic variant hereinafter); all of them were classified as (likely) pathogenic following the American College of Medical Genetics and Genomics and the Association for Molecular Pathology (ACMG/AMP) guidelines [25] and were confirmed by Sanger sequencing. 

### 2.3. Variant Nomenclature

Human Genome Variation Society (HGVS)-approved guidelines [26] were used for *BARD1* variant nomenclature using NM_000465.2 (LRG_297). For variant numbering, nucleotide 1 is the A of the ATG translation initiation codon.

### 2.4. Co-Segregation Analysis and Loss of Heterozygosity (LOH)

Both analyses were performed by Sanger sequencing when samples from relatives or tumor DNA were available.

### 2.5. gnomAD Analysis

The Genome Aggregation Database (gnomAD) non-Finnish European population, non-cancer dataset (v2.1.1) [27] was used as a control population. Variants were downloaded and filtered to identify predicted loss-of-function variants in *BARD1*. CNV screening was performed in the gnomAD SVs v2.1 dataset.

### 2.6. Statistical Analysis

Differences in allele frequency between cases and controls were determined by the Fisher exact test. Odds ratios (OR) and the corresponding 95% confidence intervals (CI) were determined for two-by-two comparisons. Statistical tests were carried out using R v.3.5.1.

## 3. Results

In our study cohort of 4015 unrelated patients with hereditary breast and/or ovarian cancer, 476 PVs were identified as per clinical gene panel analysis (Table 1), representing 11.86% patients harboring PVs in high- to moderate-penetrance BC/OC-associated genes. In addition, with the aim of investigating the role of PVs in the *BARD1* gene, we performed an exhaustive analysis of truncating, splicing and CNVs in this gene. Nineteen patients carried heterozygous germline PVs in *BARD1*, resulting in a carrier frequency of 0.47%. Among them, one patient additionally carried a PV in the HBOC-predisposing gene *BRCA2* (patient 10; *BRCA2* c.3264dupT; p.(Gln1089Serfs*10)) (Table 2). The remaining 18 *BARD1*-mutated index patients tested negative for PVs in other BC/OC genes (for more details of the genes analyzed according to the phenotype, refer to Feliubadaló et al., 2019 [24]). Thus, after excluding carriers of other HBOC PVs, the global *BARD1* carrier frequency throughout our cohort of patients was 0.45%. The percentage of deleterious *BARD1* variants in the subset of patients with hereditary breast cancer (HBC) was 0.50%, 0.42% in hereditary ovarian cancer (HOC) cases and 0.33% in patients with HBOC (Table 1). No *BARD1* PVs were identified in our set of 194 cancer-free individuals. In order to increase the control cohort, loss-of-function *BARD1* variants were screened in the non-Finnish European gnomAD 2.1.1 (non-cancer) population, identifying a total of 61 heterozygous carriers out of 51,202 individuals (0.12%). The comparison of carrier frequencies between the patient and control cohorts revealed an overall significant association of *BARD1* PVs (OR = 3.78; CI = 2.10–6.48; *p* = 1.16 × 10^−5^). This association was also significant in the HBC group (OR = 4.18; CI = 2.10–7.70; *p* = 5.45 × 10^−5^). Moreover, deleterious *BARD1* variants demonstrated an increased risk in the HOC and HBOC groups, although the differences did not reach statistical significance (OR = 3.53, CI = 0.71–10.86, *p* = 0.06 and OR = 2.77, CI = 0.33–10.47, *p* = 0.17, respectively) (Table 1). 

The clinical phenotype of *BARD1*-mutated patients is depicted in Table 2. Sixteen developed BC at a median age of 41 years (27–63), younger than the general population (median age at diagnosis 62 years old in females, according to NCI’s SEER 21 2013–2017 Program). Of these, 10 were diagnosed with at least one TNBC. We compared the prevalence of deleterious *BARD1* variants between women diagnosed with TNBC and other BC subtypes and found significant differences according to the triple-negative status of carriers. deleterious *BARD1* variants were enriched in HBC families where the index case developed TNBC (OR = 5.40; CI = 1.77–18.15; *p* = 0.001) (Table 3). Regarding OC cases, three patients were diagnosed at a median age of 62 years (59–62)—two were diagnosed with high-grade ovarian serous carcinoma (HGOSC) and one with endometrioid carcinoma (EC).

**Figure 1:**
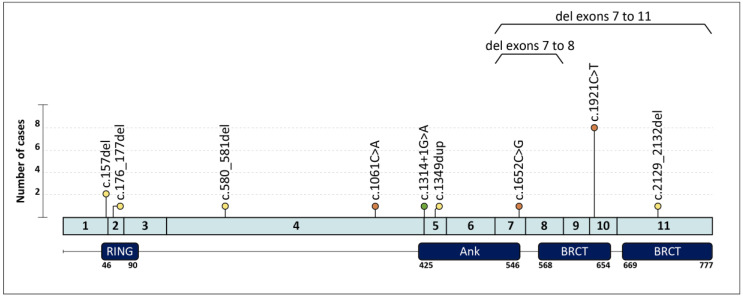
Spectrum of *BARD1* germline pathogenic variants found in our cohort. Locations of variants are displayed by lollipop structures with the following color code: orange for nonsense variants, yellow for frameshift variants and green for splicing variants. Horizontal lines correspond to copy number variants, each found in one index case. The different BARD1 protein domains are shown in dark blue boxes with an amino acid numbered scale.

Two recurrent variants were identified in our set of samples. *BARD1* c.1921C > T; p.(Arg641*) was found in eight unrelated patients, thus representing the most frequent variant in our cohort. Besides, two unrelated patients harbored the *BARD1* c.157del; p.(Cys53Valfs*5) variant. The nine remaining variants were identified in one index case each (Figure 1). It is worth mentioning that we performed RT-PCR analysis of the splicing variant c.1314+1G > A, which causes skipping of exons 3 and 4 (r.216_1314del; p.(Ser72Argfs*37)) (data not shown). Interestingly, we identified two copy number variants (CNVs) (Table 2). One consisted in the deletion of exons 7 and 8, which was experimentally validated by RT-PCR analysis in the proband’s cDNA (data not shown). This variant causes an out-of-frame deletion predicted to generate a truncated protein. The other CNV involved the loss of exons 7 to 11 and was validated using an MLPA custom probe. This deletion would presumably result in a BARD1 protein lacking both BRCT domains and the C-terminal region of the Ank domain. The screening of CNVs in the Genome Aggregation Database (gnomAD) splicing variants (SVs) dataset did not identify any CNV in the control population.

Regarding co-segregation and LOH studies, in a previous publication by our group, we reported the results of the co-segregation of family 16 [29]: the proband’s mother, diagnosed with BC, as well as the sister and the maternal cousin, both affected by BC, had the same *BARD1* variant; the variant was also found in the proband’s 39-year-old daughter, although she was asymptomatic. In the rest of the families, the co-segregation study was scarcely informative. In family 1, the proband’s cousin was diagnosed with peritoneal carcinoma (PC) at age 73 and harbored the same *BARD1* PV. In families 4 and 15, the probands inherited the *BARD1* PV from their respective mothers, also affected by BC. However, in families 13 and 14, the probands inherited the *BARD1* PV from asymptomatic mothers. LOH analysis could only be performed in a tumor sample from the proband in family 14, but there was no evidence of LOH.

## 4. Discussion

In the present study, we performed a comprehensive analysis of the *BARD1* gene in a cohort of 4015 hereditary BC/OC patients. The screening for germline PVs evidenced that *BARD1* heterozygous carriers have an overall increased risk (OR = 3.78; CI = 2.10–6.48; *p* = 1.16 × 10^−5^). When stratified by clinical suspicion, the estimated risk for HBC patients resulted in a significant OR = 4.18 (CI = 2.1–7.7; *p* = 5.45 × 10^−5^). These results are comparable to those previously reported by several case–control studies. The largest analysis to date was performed by Couch et al. in a cohort of 28,536 BC patients, proposing *BARD1* as a moderate-risk gene with an OR = 2.16 (CI = 1.31–3.63; *p* = 2.26 × 10^−3^) [9]. Similarly, Slavin et al. reported an OR = 3.18 (CI = 1.34–7.36; *p* = 0.012) [10] and Weber-Lassalle et al. reported an OR = 5.35 (CI = 3.17–9.04; *p* < 0.00001) [11] in 2134 and 4469 familial BC patients, respectively. Besides, a recent meta-analysis by Suszynska and Kozlowski collected data from a total of 123 published studies and consistently reported an OR = 2.90 (CI = 2.25–3.75; *p* < 0.0001) over a cumulative cohort of ~48,700 BC patients [30]. However, there are some studies that failed to identify a significant association with BC risk, such as those published by Castéra et al. and Lu et al. [15,16].

An increase in the prevalence of PVs in *BARD1* among TNBC patients has been repeatedly suggested [12,13,31,32]. In agreement with this hypothesis, we identified ten *BARD1* PV carriers from 680 TNBC cases (carrier frequency = 0.9%), resulting in an OR = 5.40 (CI = 1.77–18.15; *p* = 0.001). Our results are comparable to the analysis of 4090 TNBC cases performed by Shimelis et al., who identified 25 individuals harboring *BARD1* PVs (0.61%) and obtained an OR = 5.92 (CI = 3.36–10.27; *p* = 2.20 × 10^−9^) [14], whereas a surprisingly high OR = 11.27 (CI = 3.37–25.01) was reported by Castéra et al. [15]. Despite the reduced sample size of our subset of TNBC patients, our results support that deleterious *BARD1* variants were enriched in TNBC cases. Further studies in larger cohorts will be necessary to more precisely assess the *BARD1*-associated risk with this tumor phenotype.

Our results also showed a trend, although non-significant, for HOC patients (OR = 3.53). Previous studies focusing on *BARD1* as an OC-predisposing gene have shown inconsistent results. Only Norquist et al. revealed a significant OR = 4.2 (CI = 1.4–12.5; *p* = 0.02) in 1915 OC cases [18], similar to that reported in our set of samples. Contrarily, the analysis of 3261 epithelial OC cases by Ramus et al. and 6294 OC cases by Lilyquist et al. resulted in non-significant associations of deleterious *BARD1* variants with OC risk [17,19]. The meta-analysis by Suszynska and Kozlowski could not detect an association of *BARD1* with OC risk in a cumulative set of ~20,800 OC cases either [30]. 

Unraveling the contribution of moderate-penetrance genes to HC predisposition is challenging, as the low incidence of PVs detected in these genes results in inaccurate estimates of their associated risks. Due to the limited number of carriers identified, increasing the study size is mandatory to improve the statistical power. Besides, case–control studies usually rely on controls from publicly available databases to reach statistical power instead of using geographically matched controls (GMCs), potentially causing an overestimation of the calculated ORs [9]. Multi-centric international studies could potentially reduce this heterogeneity by defining common inclusion criteria for patients and harmonizing the methodological features. It is also very likely that the true prevalence of *BARD1* PVs has been underrated. As a consequence of the lack of functional assays, we have not contemplated missense, synonymous and intronic variants in the risk calculations, as we cannot be certain of their pathogenicity.

It is worth emphasizing that we have performed a screening of CNVs in our cohort of HC patients, resulting in the identification of two large deletions (exons 7 to 8 and exons 7 to 11), accounting for 10.5% of the PVs. To our knowledge, only a small fraction of published studies have also performed this analysis and only seven CNVs have been identified so far: exon 1 deletion [33], exon 2 deletion [34], exon 1 to 6 deletion [35], exon 5 to 7 deletion [36], exon 8 to 11 deletion [37] and two whole-gene deletions [37,38]. While no CNVs were identified in the gnomAD SV control population dataset, analysis of *BARD1* CNVs in HC cohorts is strongly recommended considering the significant contribution in our series of this kind of variant.

*BARD1* has been included in multi-gene panels since it was regarded as a potential cancer-predisposing gene [39], despite the lack of robust risk estimates. The identification of *BARD1* PV carriers should be taken with caution, as inherited PVs in moderate- to low-penetrance genes may not necessarily be responsible for all the cancer diagnoses in a family. Nevertheless, although the clinical evidence available to date is still insufficient to impact risk management, continued testing of *BARD1* will permit access to the carrier status once recommendations for *BARD1* PV carriers become available in the future.

Taken together, our results confirm *BARD1* as a BC susceptibility gene and highlight a stronger association with triple-negative tumors. Future studies aimed at screening larger cohorts and refining the classification of *BARD1* variants will help to elucidate its role as a breast and/or ovarian cancer gene as well as define medical recommendations for *BARD1* PV carriers.

## Figures and Tables

**Table 1:** Summary of the next-generation sequencing (NGS) panel results in our hereditary breast and/or ovarian cancer (HBOC) cohort and in the control populations.

Clinical Indication	Number of Patients (%)	Genes Tested by Phenotype	Number of PVs (%)	*BARD1* (%)	*BARD1* Excluding Patients with Other PVs (%)
Only Hereditary Breast Cancer, HBC	2622 (65.31%)	*ATM, BRCA1, BRCA2, CHEK2, MLH1, MSH2, MSH6, PALB2, TP53*	270 PVs (10.30%): *ATM* (34)*, BRCA1* (71)*, BRCA2* (90)*, CHEK2* (27)*, MLH1* (3)*, MSH2* (1)*, MSH6* (2)*, PALB2* (37)*, TP53* (5)	13 (0.50%) OR = 4.18 (2.10–7.70) **p = 5.45 × 10^−5^	13 (0.50%) OR = 4.18 (2.10–7.70) **p = 5.45 × 10^−5^
Only Hereditary Ovarian Cancer, HOC	715 (17.81%)	*BRCA1, BRCA2, BRIP1, MLH1, MSH2, MSH6, RAD51C, RAD51D*	93 PVs (13.01%): *BRCA1* (39)*, BRCA2* (35)*, BRIP1* (6)*, MLH1* (1)*, MSH6* (4)*, RAD51C* (4)*, RAD51D* (4)	3 (0.42%) OR = 3.53 (0.71–10.86) *p* = 0.06	3 (0.42%) OR = 3.53 (0.71–10.86) *p* = 0.06
Hereditary Breast and Ovarian Cancer, HBOC	608 (15.14%)	*ATM, BRCA1, BRCA2, BRIP1, CHEK2, MLH1, MSH2, MSH6, PALB2, RAD51C, RAD51D, TP53*	104 PVs (17.11%): *ATM* (7)*, BRCA1* (45)*, BRCA2* (32)*, BRIP1 (7), CHEK2* (6)*, MSH2* (1)*, PALB2* (3)*, RAD51C* (1)*, RAD51D (1), TP53* (1)	3 (0.49%) OR = 4.16 (0.83–12.79) * *p* = 0.04	2 (0.33%) OR = 2.77 (0.33–10.47) *p* = 0.17
HBC/HOC/HBOC + Other clinical indications	70 (1.74%)	Details in Ref: [24]	9 PVs (12.86%): *ATM* (2), *BRCA1* (2), *BRCA2* (1), *MSH6* (2), *PTEN* (1), *RAD51C* (1)	0 (0%)	0 (0%)
Total	4015		476 (11.86%)	19 (0.47%) OR = 3.99 (2.25–6.77) ** *p* = 3.48 × 10^−6^	18 (0.45%) OR = 3.78 (2.10–6.48) ** *p* = 1.16 × 10^−5^
			Controls studied		
			Spanish population cohort (*n* = 194)		0 (0%)
			gnomAD non-Finnish European, non-cancer cohort (*n* = 51,202)		61 (0.12%)

PV: pathogenic variant; OR: odds ratio. * α < 0.05. ** α < 0.01.

**Table 2:** Genotype and phenotype data of index patients carrying heterozygous germline pathogenic variants in the BRCA1-associated ring domain 1 (*BARD1)* gene.

Family	Clinical Indication	Cancer Type (Age at dx)	Tumor Phenotype	Family History (Age at dx)	*BARD1* PV (c.)	*BARD1* PV (p.)	Additional PVs
1	HBC	Breast (40,58)	ILC ER+ Her2-; TNBC	Cousin: PC (73)	c.157del	p.(Cys53Valfs*5)	
2	HBOC	Breast (30)	IDC ER+ Her2-				
3	HOC	Ovary (59)	HGOSC		c.176_177del	p.(Glu59Alafs*8)	
4 ^†^	HBC	Breast (27,42)	ER+ BC; TNBC	Mother: Breast (44,44)	c.580_581del	p.(Arg194Glyfs*2)	
5	HBC	Breast (38)	IDC ER+ Her2-	Aunt: Breast (37) ^‡^, Aunt: Breast (36)	c.1061C > A	p.(Ser354*)	
6	HOC	Ovary (62)	EC		c.1314+1G > A	p.?	
7	HBC	Breast (49)	TNBC		c.1349dup	p.(Asn450Lysfs*4)	
8	HBC	Breast (31)	TNBC	Aunt: Breast (64) ^‡^, Aunt: Breast (64) ^‡^	c.1652C > G	p.(Ser551*)	
9	HBC	Breast (56)	TNBC		c.1921C > T	p.(Arg641*)	
10	HBOC	Breast (54)	IDC ER+ Her2-	Mother: Ovary (63)			*BRCA2* c.3264dupT; p.(Gln1089Serfs*10)
11	HBC	Breast (63)	TNBC	Aunt: Breast (60) ^‡^, Cousin: Breast (54) ^‡^			
12	HBC	Breast (40)	IDC ER+ Her2+	Mother: EC (62), Breast (64)			
13	HBC	Breast (49)	TNBC				
14	HBC	Breast (30)	TNBC				
15	HBC	Breast (46,56,56)	IDBC; bilateral IDBC	Mother: Breast (78)			
16 ^^^	HBC	Breast (40,47)	TNBC; TNBC	Sister: Breast (46); Sister: Breast (48); Mother: Breast (48); Cousin: Breast (46)			
17	HBOC	Breast (42)	IDC ER+ Her2-	Uncle: Breast (71); Aunt: Ovary (62)	c.2129_2132del	p.(Asp710Valfs*3)	
18	HOC	Ovary (62)	HGOSC		c.(1568+1_1569-1)_(1810+1_1811-1)del Exons 7–8 deletion		
19	HBC	Breast (44)	TNBC	Mother: Breast (69); Aunt: Breast (60)	g.(?_215617227)_(215593730_?) Exons 7–11 deletion		

HBC: hereditary breast cancer; HBOC: hereditary breast and ovarian cancer; HOC: hereditary ovarian cancer; Dx: diagnosis; PV: pathogenic variant; HGOSC: high-grade ovarian serous carcinoma; PC: peritoneal carcinoma; EC: endometrioid carcinoma; BC: breast cancer; ILC: invasive lobular carcinoma of the breast; IDC: invasive ductal carcinoma of the breast; IDBC: intraductal breast carcinoma; TNBC: triple-negative breast cancer; ER: estrogen receptor status; Her2: human epidermal growth factor receptor 2 status. ^‡^ Cancer diagnosis unconfirmed. ^†^ Results previously reported in Ref [28]; ^^^ Results previously reported in Ref [29].

**Table 3:** Summary of the triple-negative status of the hereditary breast cancer cohort.

Group	Number of Patients	*BARD1*-Mutated
TNBC patients	680	10 (0.88%) OR = 5.40 (1.77–18.15) *p* = 0.001 **
Non-TNBC patients	2179	6 (0.28%)
Total	2859	16

TNBC: triple-negative breast cancer; OR: odds ratio. ** α < 0.01.

## Data Availability

Data is contained within the article.
